# Prothrombin Complex Concentrate for Rapid Reversal of Warfarin Anticoagulation to Allow Neuraxial Blockade

**DOI:** 10.1155/2014/126864

**Published:** 2014-03-04

**Authors:** Conor Skerritt, Stephen Mannion

**Affiliations:** Department of Anaesthesia, South Infirmary, Victoria University Hospital, Old Blackrock Road, Cork, Ireland

## Abstract

The development of Prothrombin Complex Concentrates (PCCs) has led to better outcomes in patients receiving emergency reversal of warfarin. However, most published data describes the use of PCCs in the setting of major bleeding or emergent major surgery, with little information on neuraxial blockade. We describe a case of rapid warfarin reversal using PCC and subsequent surgery under spinal anaesthesia in an 87-year-old lady, for whom general anaesthesia was deemed high risk. Her international normalised ratio (INR) on the morning of surgery was 1.8, precluding neuraxial blockade; however, it was felt that given, the need for imminent surgery, immediate reversal of the warfarin was indicated. We administered a single dose of 23 units/kg PCC and 5 mg vitamin K. Her INR 1 hour following PCC was 1.2, and spinal anesthetic was administered. The patient then underwent excision of melanoma deposits from her leg and groin dissection. There were no complications, the patient recovered satisfactorily, and there were no thrombotic or hemorrhagic events at 30 days postoperatively. This case study demonstrates a novel use of PCCs; in certain patients, PCCs may be safely used for immediate reversal of warfarin to allow for neuraxial blockade, safer anaesthesia, and better outcomes.

## 1. Introduction

The development of Prothrombin Complex Concentrates (PCCs) has led to better outcomes in patients receiving emergency reversal of warfarin anticoagulation [1–4]. However, most of the published data describes the use of PCCs in the setting of major bleeding or emergent major abdominal surgery, with a dearth of information on neuraxial blockade. We describe a case of rapid warfarin reversal using PCC and subsequent surgery under spinal anaesthesia, with a good surgical outcome and no thrombotic or haemorrhagic events at 30 days.

## 2. Case/Methods

An 87-year-old lady was scheduled for urgent palliative removal of malignant melanoma and satellite lesions from her lower leg and ipsilateral groin dissection for metastatic disease. Her background history included atrial fibrillation which was rate controlled (digoxin) and required anticoagulation (warfarin). Her background also included mitral regurgitation, hypertension, and hypothyroidism. As her mobility was severely limited by osteoarthritis of the hip, it was difficult to clinically establish her exercise tolerance, and it was decided that she would be more suitable for neuraxial blockade than general anaesthesia. She was instructed to discontinue warfarin for 4 days prior to her planned surgery, which she did. However, on the day of her planned procedure, her international normalised ratio (INR) was 1.8. A decision was made to rapidly reverse her anticoagulation and proceed with the surgery under spinal anaesthesia. Postponing her surgery would have caused a delay of a number of days, due to the specifics of scheduling her case, and it was felt that, given the existing tumour burden, a delay of this magnitude was not acceptable.

A decision was made that rapid reversal of anticoagulation was needed and that, based on current evidence in medical literature, a combination of intravenous vitamin K and PCC would be most effective [5]. 5 mg intravenous vitamin K was given as a slow bolus, and using the hospital's protocol regarding the administration of blood products, 1500 units of Octaplex (PCC containing factors II, VII, IX, X, and proteins C and S) were given over 35 minutes. The total volume of this solution was 60 mL. The patient's INR was rechecked 1 hour following completion of the PCC infusion and was found to be 1.2, within the acceptable recommended range (i.e., <1.5) [6]. A single shot spinal anaesthesia was performed by an experienced anesthesiologist, with the patient in the sitting position. Using an *L*_4/5_ paramedian approach and a 22 G spinal needle, 1.5 mL 0.5% heavy bupivacaine and 25 *μ*g of fentanyl were injected intrathecally in an atraumatic fashion (first pass, CSF identified, and no blood seen).

## 3. Results

The block was then clinically evaluated, and, finding good blockade (loss of temperature sensation at *T*_10_), surgery was commenced. The patient did not receive any hypnotic or sedative medication in the perioperative period.

The surgery carried out was a right sided groin dissection, excision of melanoma deposits from right lower leg, split skin graft, and diathermy ablation of melanoma satellite lesions. The surgery was uncomplicated, intraoperative blood loss was minimal (<250 mL), and the sensory blockade was adequate. The duration of surgery was approximately 100 minutes.

Examination of the patient at 60 minutes postoperatively (160 minutes after spinal) revealed return of gross motor function to both lower limbs and sensation at *T*_10._ Further examination at 250 minutes postoperatively (310 minutes after spinal) revealed full return of motor function and full sensation in both lower limbs, with a pain score of 3/10 on oral analgesics. The patient received her usual dose of warfarin that evening.

At 30-day followup, there were no thrombotic or haemorrhagic events, the surgical wounds were satisfactory, and the skin graft was intact.

## 4. Discussion

Prothrombin Complex Concentrates (PCCs) are pooled plasma products which contain a mixture of vitamin K dependent clotting factors. Originally designed for the treatment of haemophilias, they are now used for reversal of coagulopathies of the vitamin K dependent pathways, both congenital and acquired or iatrogenic. PCC has been used to rapidly correct various abnormalities of coagulation in patients with haemorrhage or the need for immediate surgical or invasive medical intervention [1–4]. It has been found to be superior to vitamin K alone in the reversal of the effects of warfarin [7]. However, there is little published evidence regarding the use of PCC for reversal of warfarin prior to spinal anaesthesia or other forms of neuraxial anaesthesia. Single shot spinal anaesthesia in the presence of coagulopathy has been classified as “very high risk” by the American Society of Regional Anaesthesia (ASRA), the European Society of Regional Anaesthesia (ESRA), and the Association of Anaesthetists of Great Britain and Ireland (AAGBI) and, as such, should not be attempted with an INR >1.4.

The optimal dose of PCC for the rapid reversal of warfarin has been extensively investigated, and numerous data on the subject have been published [7–9]. Mean doses in these articles of research have ranged from 12 units/kg to 28.5 units/kg. The recommendations of the manufacturer are as follows [10]: in a patient whose INR is 2–2.5, normalisation of INR (i.e., INR ≤1.2) within 1 hour may be achieved with a single dose of 0.9–1.3 mL/kg, which equates to 22.5–32.5 units/kg. However, our patient's initial INR was 1.8, and the manufacturer's published data did not refer to INRs of <2.0. Therefore, the manufacturer's recommendations were used as guidelines and interpreted alongside other published data. Our patient received a single dose of 23 units/kg. This not only reflects the data we extrapolated from the manufacturer's guidelines, but also reflects the more recently published articles on the matter.

The usefulness of contemporaneous vitamin K with PCC to achieve more timely reversal of anticoagulation has also been described [5], and it is for this reason that we included it as part of our treatment.

The rapid reversal of anticoagulation will, of course, predispose the patient to thrombotic events (stroke, coronary ischemia, peripheral ischemia, and disseminated intravascular coagulation), but the likelihood of these depends also on the initial indication for anticoagulation, as well as the dose of PCC given. Other potential adverse effects include hypersensitivity, headache, transmitted viral illnesses, and transient hepatic transaminitis. Our patient was anticoagulated because she had atrial fibrillation, and although her relative risk of a thrombotic event was high if she was not anticoagulated [11], a brief window of normal INR was estimated to be low risk, and, indeed, at 30 days after intervention, there were no thrombotic events in our case study.

## 5. Conclusion

In conclusion, our case study demonstrates that Prothrombin Complex Concentrate (PCC), as a single dose of 23 units/kg, can be used to acutely reverse the anticoagulant effect of warfarin, such that effective spinal anaesthesia may be safely administered. The novel usage of this well-established product means that neuraxial blockade may be administered in cases where the risk of bleeding complications would previously have precluded its use, thus allowing for safer anaesthesia and better outcomes for our patients.
